# Effects of intensive blood pressure lowering on mortality and cardiovascular and renal outcomes in type 2 diabetic patients: A meta-analysis

**DOI:** 10.1371/journal.pone.0215362

**Published:** 2019-04-12

**Authors:** Jing Wang, Yalei Chen, Weihao Xu, Nianfang Lu, Jian Cao, Shengyuan Yu

**Affiliations:** 1 Department of Neurology of The First Medical Center, Chinese PLA General Hospital, Beijing, China; 2 School of Medicine, Nankai University, Tianjin, China; 3 Department of Critical Care Medicine, Beijing Electric Power Hospital, Beijing, China; 4 Geriatric Cardiology Department of The Second Medical Center & National Clinical Research Center for Geriatric Diseases, Chinese PLA General Hospital, Beijing, China; University of Oxford, UNITED KINGDOM

## Abstract

**Background:**

Previous studies have demonstrated that intensive blood pressure (BP) lowering treatment reduces the risk of all-cause mortality and provides greater vascular protection for patients with hypertension. Whether intensive BP lowering treatment is associated with such benefits in patients with type 2 diabetes mellitus remain unknown. We aimed to clarify these benefits by method of meta-analysis.

**Methods:**

The PubMed, EMBASE, Science Citation Index and Cochrane Library databases were searched to identify randomized controlled trials (RCT) that fulfilled study inclusion criteria. Two investigators independently extracted and summarized the relevant data of the included trials. Random-effects model was applied to calculate the estimates of all effect measures.

**Results:**

We included 16 RCTs and our meta-analysis showed that intensive BP lowering treatment vs less intensive BP lowering treatment resulted in significant reductions in the all-cause mortality risk [relative risk (RR), 0.82; 95% CI, 0.70–0.96], major CV events (RR, 0.82; 95% CI, 0.73–0.92, MI (RR, 0.86; 95% CI, 0.77–0.96), stroke (RR, 0.72; 95% CI, 0.60–0.88, CV death (RR, 0.73; 95% CI, 0.58–0.92) and albuminuria progression (RR, 0.91 95% CI, 0.84–0.98). However, intensive BP lowering treatment had no clear effect on non-CV death (RR, 0.97; 95% CI, 0.79–1.20), heart failure (HF) (RR, 0.88; 95% CI, 0.71–1.08) or end-stage kidney disease (ESKD) (RR, 1.00; 95% CI, 0.75–1.33). Subgroup analysis showed that the reduction in all cause-mortality was consistent across most patient groups, and intensive BP lowering treatment had a clear benefit even in patients with systolic blood pressure lower than 140 mm Hg. However, the benefit differed in patients with different CV risk (≥10%: RR, 0.77, 95%CI, 0.64–0.91; <10%: RR, 1.04, 95%CI, 0.84–1.29; *P*_hetero_ = 0.028).

**Conclusions:**

Our data indicate that intensive BP lowering treatment provides greater benefits than less intensive treatment among patients with type 2 diabetes mellitus. Further studies are required to more clearly evaluate the benefits and harms of BP targets below those currently recommended with intensive BP lowering treatment.

## Introduction

Diabetes mellitus (DM) is a global public health problem. DM is estimated to affect 116 million Chinese people, according to a recent epidemiological survey[1], and is estimated to affect 400 million individuals worldwide by the year of 2030[2]. People with DM are at high risk of cardiovascular (CV) events, such as stroke and myocardial infarction (MI), and all-cause mortality at any level of blood pressure (BP)[3–5].

Current guidelines regarding the BP target in diabetic patients are inconsistent. The 2013 European Society of Hypertension/European Society of Cardiology (ESH/ESC) Task Force stopped recommending the lowering of BP to <130/80 mm Hg in patients with diabetes, and recommended a goal of <140/90 mm Hg, in contrast to the 2007 ESH/ESC guideline[6,7]. The Eighth Joint National Committee on the Prevention, Detection, Evaluation, and Treatment of High Blood Pressure (JNC 8) has recommended a treatment goal of <140/90 mm Hg for diabetes patients aged ≥18 years[8]. However, the latest 2018 Canadian hypertension guideline and 2017 American College of Cardiology/American Heart Association (ACC/AHA) guideline have both recommended that adults with DM be treated to attain <130/80 mm Hg[9, 10].

Several randomized controlled trials (RCTs) have evaluated the effects of intensive BP lowering treatment on DM patients, with some studies showed lower risk of all-cause mortality and greater vascular protection from intensive treatment, while others indicated no benefit [11–26]. Previous systematic reviews have explored the effects of intensive BP lowering treatment and the optimal achieved BP level in diabetes patients[27–31], but these reviews focused on fewer clinical outcomes (major CV events, MI and stroke)[27, 28] and included many RCTs that aimed only to investigate the drug effects or compare the effects of two or more drugs rather than to explore the effects of intensive BP lowering treatment or the optimal BP level in diabetes patients[29–31]. Therefore, our objective was to conduct a new systematic review and meta-analysis that only included RCTs aimed at evaluating the effects of intensive BP lowering treatment to investigate whether more intensive BP control compared with less intensive BP control was associated with a reduced mortality risk and better CV and renal outcomes in diabetic patients.

## Methods

### Data sources and searches

This study was performed according to the recommendation of the Preferred Reporting Items for Systematic Reviews and Meta-analysis (PRISMA) guidelines (checklist is shown in S1 Table) [32]. The literature search was performed in May 2018. We systematically searched the PubMed, EMBASE, Science Citation Index and Cochrane Library databases from January 1, 1950, to May 1, 2018. The references of the identified publications, previous systematic reviews and guidelines in the discipline were also manually searched for additional studies. No language restrictions were applied. Studies were restricted to RCTs, clinical trials or controlled clinical trials. The detailed literature search strategies are provided in S1 Appendix.

### Study selection

We included RCTs comparing different BP lowering treatment arms (more intensive vs. less intensive BP control or active BP lowering treatment vs. placebo treatment) in type 2 diabetic patients who were older than 18 years. RCTs with a DM subgroup could also be included. If no relevant data for the DM subgroup were provided in the literature, we emailed the trial investigators and requested the data of the DM subgroup. The follow-up period of the included RCTs was at least 12 months. We excluded strictly comparative trials, evaluating one agent against another, as well as trials with combined interventions.

### Data extraction and quality assessment

Two investigators (JW and YLC) independently extracted and summarized the relevant data of the included trials. The following information was extracted from each included study: the first author’s name (trial abbreviation), publication year, study design, inclusion criteria and patient population in each trial, proportion of female participants, mean age of the study population, median follow-up time in years, estimated CV risk after 10 years, medications used by the different groups, baseline BP level, BP targets in the different groups, achieved BP level in the intensive group, achieved BP level in the less intensive group and difference in BP reduction. Any disagreements regarding the extracted data were first discussed by the two investigators (JW and WHX). If a consensus was not reached, the discrepancies were resolved by a third investigator (JC). The methodological quality of the included trials was evaluated with the Cochrane risk of bias tool[33].

The primary outcome was all-cause mortality. Other outcomes of interest included CV outcomes and renal outcomes. CV outcomes included major CV events, CV death, non-CV death, MI, stroke, and heart failure (HF). Renal outcomes included end stage kidney disease (ESKD) and albuminuria progression.

### Statistical analysis

For studies that provided the relative risk (RR) and 95% confidential interval (CI) for study outcomes, we extracted these data directly. For studies that did not provide the RRs and 95% CIs, we calculated these data before pooling or extracted the relevant data from a previous meta-analysis[29–31]. The chi-square test and I-squared (*I*^2^) statistic were used to explore heterogeneity among the included studies[34]. Random-effects model was applied to calculate the estimates of all effect measures. Subgroup analysis was performed according to the study type (2 defined BP arms vs. an active group vs. placebo), whether hypertension was present in the diabetic patients, the mean age of the study population, CV risk (calculated as the incidence rate of CV death in the group receiving less intensive treatment or in the placebo control group; the observed CV death rate was extrapolated to a period of 10 years to fit the usual expression of CV risk)[35], baseline systolic BP (SBP), achieved SBP in the intensive or active group, and the SBP difference. Univariate meta-regression was performed to explore potential sources of heterogeneity. Sensitivity analysis was also performed to verify the robustness of the overall results. Potential publication bias was assessed by visual inspection of the funnel plot for all outcomes separately. In addition, Egger’s regression test[36] and Begg’s test[37] were used to statistically assess publication bias. A two-sided *P* value <0.05 was regarded as statistically significant. All analyses were performed using Stata release 12 (Stata Corp, College Station, TX, USA).

## Results

### Literature search

**Fig 1:**
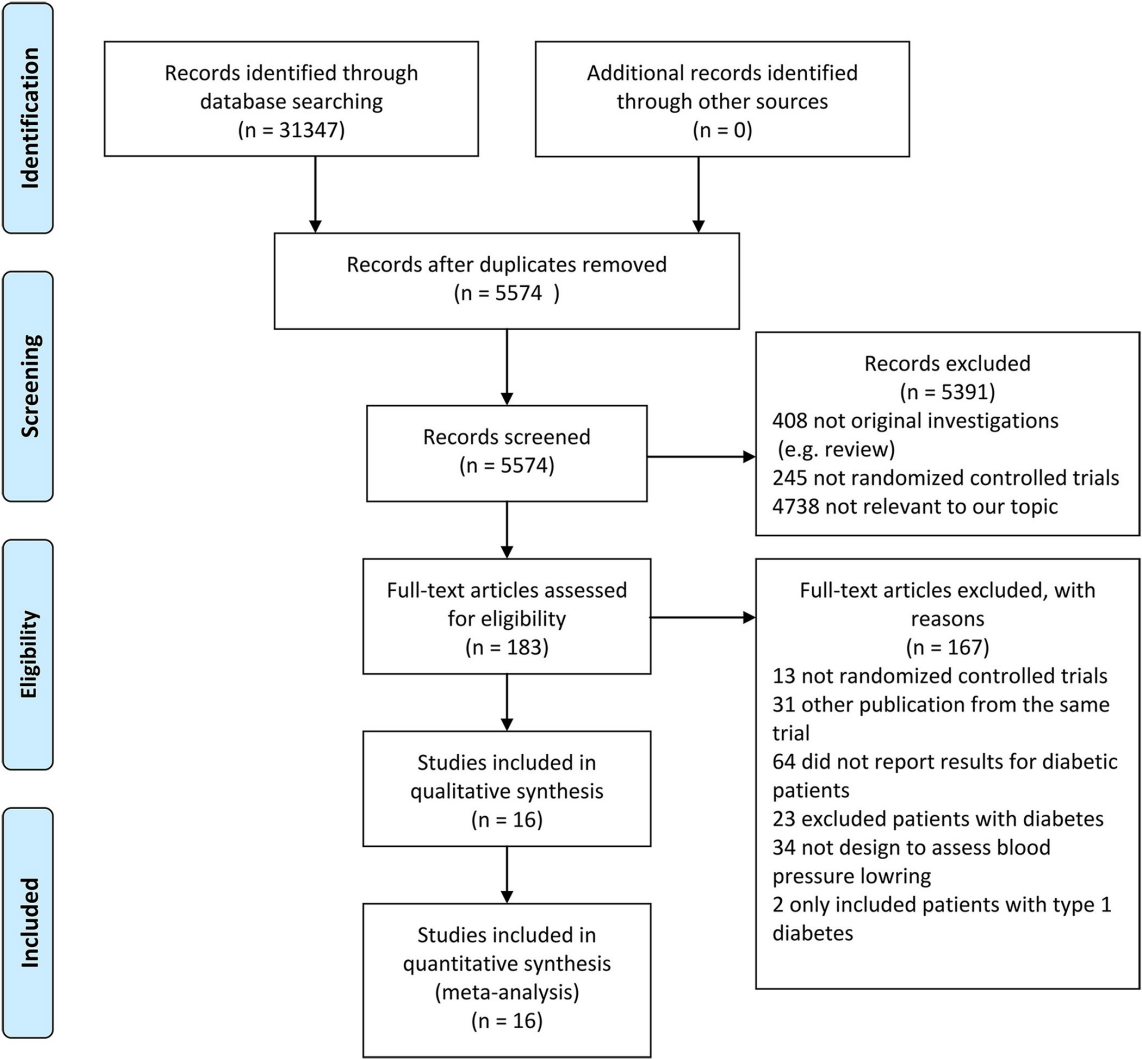
Flowchart of trial identification for meta-analysis.

A total of 31,347 articles were identified through literature searches. After removing duplicated articles, 5574 remained for further screening. Two investigators (WHX and JW) carefully read the titles and abstracts of the remaining articles and excluded another 5391 articles. Next, 183 articles were screened in a full-text review, and an additional 167 were excluded. The remaining 16 articles corresponded to 16 RCTs which satisfied the inclusion criteria. The flowchart of trial identification is shown in Fig 1.

### Study characteristics and quality assessment

Overall, 16 RCTs corresponding to 24,444 type 2 diabetic participants fulfilled the inclusion criteria. The clinical characteristics of the included trials are summarized in Table 1 and Table 2. Ten trials were designed to compare intensive BP control to less intensive BP control, and 6 trials were designed to compare active treatment to placebo treatment. Of the 16 included trials, 2 trials had all with hypertension and all with DM, 2 trials had some hypertensive and some normotensive and all with DM, 2 trials had all normotensive and all with DM, 8 trials had all with hypertension and some with DM, and 2 trials had some hypertensive and some normotensive and some with DM. The mean baseline BP of the included patients was 158.3/88.0 mm Hg before treatment. After randomization and treatment, the mean achieved BP in the intensive BP lowering treatment arm was 136.6/76.7 mm Hg, and the mean achieved BP in the less intensive BP lowering treatment arm was 144.9/81.1 mm Hg. The achieved difference in the mean SBP ranged from 3.4 to 16.1 mm Hg at the end of the trial. The risk of bias of the included trials was assessed with Cochrane Collaboration’s tool and is summarized in S1 and S2 Figs.

**Table 1:** Baseline clinical characteristics of trials included in the meta-analysis.

Author/Year	Design/ Country of origin	Inclusion Criteria	Patient No.(n)	Female (%)	Mean Age (years)	Follow-up (years)	Cardiovascular Risk (%)
HDFP Group (HDFP) 1979	Randomized multicenter; USA	Age 30–69 with DBP>90 mm Hg	10940 DM:772	46.0	50.8	5	12.5
Amery et al (EWPHE) 1985	Randomized multicenter; USA	Age≥60 years with SBP 160–239 mm Hg and DBP 90–119 mm Hg	840 DM:111	69.8	72	4.6	26.6
Curb et al (SHEP) 1996	Randomized multicenter; USA	Age≥60 years with isolated systolic hypertension (SBP 160–220 mm Hg and DBP < 90 mm Hg)	4736 DM: 583	49.6	70.1	4.5	17.8
UKPD Study Group (UKPDS) 1998	Randomized multicenter; UK	Newly diagnosed type 2 diabetes with hypertension	1148	44.5	56	8.4	17.7
Hansson et al (HOT) 1998	Randomized multicenter; Sweden, Italy, Canada, USA, France and Germany	Hypertension with DBP 100–115 mm Hg	18790 DM:1501	47	61.5	3.8	11.1
Tuomilehto et al (Syst-Eur) 1999	Randomized multicenter; western and eastern Europe	Age≥60 years with isolated systolic hypertension (SBP 160–219 mm Hg and DBP < 95 mm Hg)	4695 DM:492	66.8	70.2	2	33.3
Wang et al (Syst-China) 2000	Randomized multicenter; China	Age≥60 years with isolated systolic hypertension (SBP 160–219 mm Hg and DBP < 95 mm Hg)	2394 DM:98	35.6	66.5	3	47.0
Estacio et al (ABCD-H) 2000	Randomized multicenter; USA	Type 2 diabetes with DBP≥90 mm Hg	470	32.6	57.9	5	10.7
Schrier et al (ABCD-N) 2002	Randomized multicenter; USA	Type 2 diabetes with normotension(DBP 80–89 mm Hg)	480	45.5	59.1	5.3	7.4
Berthet et al (PROGRESS) 2004	Randomized multicenter; Asia, Australia and Europe	Patients with history of stroke or TIA in previous 5 years	6150 DM:761	28	64	3.9	23.7
Estacio et al (ABCD-2V) 2006	Randomized single-center, USA	Type 2 diabetic patients, 40 to 81 years of age, with SBP<140 mm Hg, DBP between 80 and 90 mm Hg	129	32.6	56.1	2	NA
ADVANCE Collaborative Group (ADVANCE) 2007	Randomized multicenter; Australia, Asia, Europe and North America	Type 2 diabetes at the age of 30 years or older, were 55 years of age or older at study entry and had evidence of elevated risk of cardiovascular disease	11140	42.5	65.8	4.3	10.7
JATOS Study Group (JATOS) 2008	Randomized multicenter; Japan	Age between 65 and 85 years with SBP >160 mm Hg	4418 DM:521	61.1	73.6	2	1.6
Ogihara et al (VALISH) 2010	Randomized multicenter; Japan	Age between 70 and 85 years with isolated systolic hypertension (SBP >160 mm Hg and DBP < 90 mm Hg)	3260 DM:399	62.5	76.1	2.9	2.8
Accord Study Group (ACCORD) 2010	Randomized multicenter; USA, Canada	Type 2 diabetic patients with 40 years older and cardiovascular disease or 55 years older with risk for cardiovascular disease	4733	47.7	62.2	4.7	5.2
SPS3 Investigators (SPS3) 2013	Randomized multicenter; North America	Age≥40 years with normotension or hypertension had lacunar stroke	3020 DM: 1106	37	63	3.7	9.9

DM, diabetes mellitus; NA, not available; In the ABCD-2V study there was no death in the control group and CV risk cannot be calculated with the system of Prof. Zanchetti

**Table 2:** Trial interventions and their effects.

Author/Year	Antihypertensive Regimens	Baseline BP(mm Hg)	BP target in Intensive Group (mm Hg)	BP target in Less Intensive Group (mm Hg)	Achieved BP in Intensive Group (mm Hg)	Achieved BP in Less Intensive Group (mm Hg)	Difference in BP Reduction (mm Hg)
HDFP Group (HDFP) 1979	SC: step 1 to step 5 with diuretic (chlorthalidone, triamterene or spironolactone), antiadrenergic drug (reserpine, methyldopa or guanethidine sulfate),vasodilator (hydralazine) or other antihypertensive drugs as needed RC: referred care	158.8/101.5	DBP <90 mm Hg for patients with DBP ≥ 100 mm Hg or 10 mm Hg reduction for DBP 90–99 mm Hg	NR	131.5/86	141.5/92	-10/-6
Amery et al (EWPHE) 1985	Active group: Hydrochlorothiazide+triamterene and methyldopa as needed Placebo group: matching placebo	186.8/101.2	NR	NR	149.5/86.4	165.6/91.7	-16.1/-5.3
Curb et al (SHEP) 1996	Active group: Chlorthalidone with a step-up to atenolol or reserpine if needed Placebo group: Placebo and any active antihypertensive drugs prescribed by private physician	170.2/75.8	SBP <160 mm Hg for those initial SBP ≥180 mm Hg; SBP reduction≥ 20 mm Hg for those initial SBP 160–179 mm Hg	NR	146.0/68.5	155.8/70.7	-9.8/-2.2
UKPD Study Group (UKPDS) 1998	Tight group: Captopril and atenolol and other agents if needed Less tight group: Avoiding ACEI and β blocker and other agents if needed	159.3/94.0	BP<150/85	BP<180/105	144/82	154/87	-10/-5
Hansson et al (HOT) 1998	Intensive group: felodipine, ACEI, β blocker and diuretic if needed Less intensive group: same as intensive group but to achieve a higher target	174.1/105.3	DBP<80	DBP<85 or 90	143.7/81	147.1/83.9	-3.4/-2.9
Tuomilehto et al (Syst-Eur) 1999	Active group: nitrendipine and combined with or replaced by enalapril, hydrochlorothiazide, or both drugs Placebo group: matching placebo	175.3/84.5	Reduce the systolic blood pressure by at least 20 mm Hg and to less than 150 mm Hg	NR	153.2/77.7	161.8/81.6	-8.6/-3.9
Wang et al (Syst-China) 2000	Active group: nitrendipine and with possible addition of captopril, hydrochlorothiazide, or both drugs Placebo group: matching placebo	172.5/93	Reduce the SBP by at least 20 mm Hg and to less than 150 mm Hg	NR	150.1/86.3	156.1/91	-6.0/-4.7
Estacio et al (ABCD-H) 2000	Intensive group: nisoldipine or enalapril Moderate group: same as intensive group but to achieve a higher target	155/98	DBP<75	DBP 80–89	132/78	138/86	-6/-8
Schrier et al (ABCD-N) 2002	Intensive group: nisoldipine or enalapril Moderate group: Placebo and required antihypertensive drugs if needed	136.4/84.4	DBP reduction 10 mm Hg from baseline	DBP 80–89	128/75	137/81	-9/-6
Berthet et al (PROGRESS) 2004	Active group: perindopril, indapamide Placebo group: matching placebo	149.5/84.5	NR	NR	136.6/74.8	146.1/79.4	-9.5/-4.6
Estacio et al (ABCD-2V) 2006	Intensive group: Valsartan, metoprolol and hydrochlorothiazide Moderate group: matching placebo and valsartan if needed	126/84	DBP <75	DBP 80–90	118/75	124/80	-6/-5
ADVANCE Collaborative Group (ADVANCE) 2007	Active group: perindopril and indapamide Placebo group: matching placebo	145/81	NR	NR	134.7/74.8	140.3/77	-5.6/-2.2
JATOS Study Group (JATOS) 2008	Strict group: efonidipine Mild group: same as strict group but to achieve a higher target	172.3/87.3	SBP<140	SBP 140–160	135.9/74.8	141.5/75.7	-5.6/-0.9
Ogihara et al (VALISH) 2010	Strict group: valsartan and other antihypertensive drugs if needed Moderate group: same as strict group but to achieve a higher target	168.0/80.7	SBP <140	SBP ≥140 to <150	136.6/74.8	140.3/75.7	-3.7/-0.9
Accord Study Group (ACCORD) 2010	Intensive group: ACEI, thiazide, β blocker, CCB, reserpine or α blocker to achieve target Standard group: same as intensive group but to achieve a higher target	139.2/76.0	SBP<120	SBP<140	119.3/64.4	133.5/70.5	-14.2/-6.1
SPS3 Investigators (SPS3) 2013	Lower target group: any drugs from major classes of antihypertensive medication to achieve settled target Higher target group: any drugs from major classes of antihypertensive medication to achieve settled target	144/77	SBP<130	SBP 130–149	125.8/69	136.8/74	-11.0/-5.0

NR, not report

### Effects of intensive BP lowering on all-cause mortality

**Fig 2:**
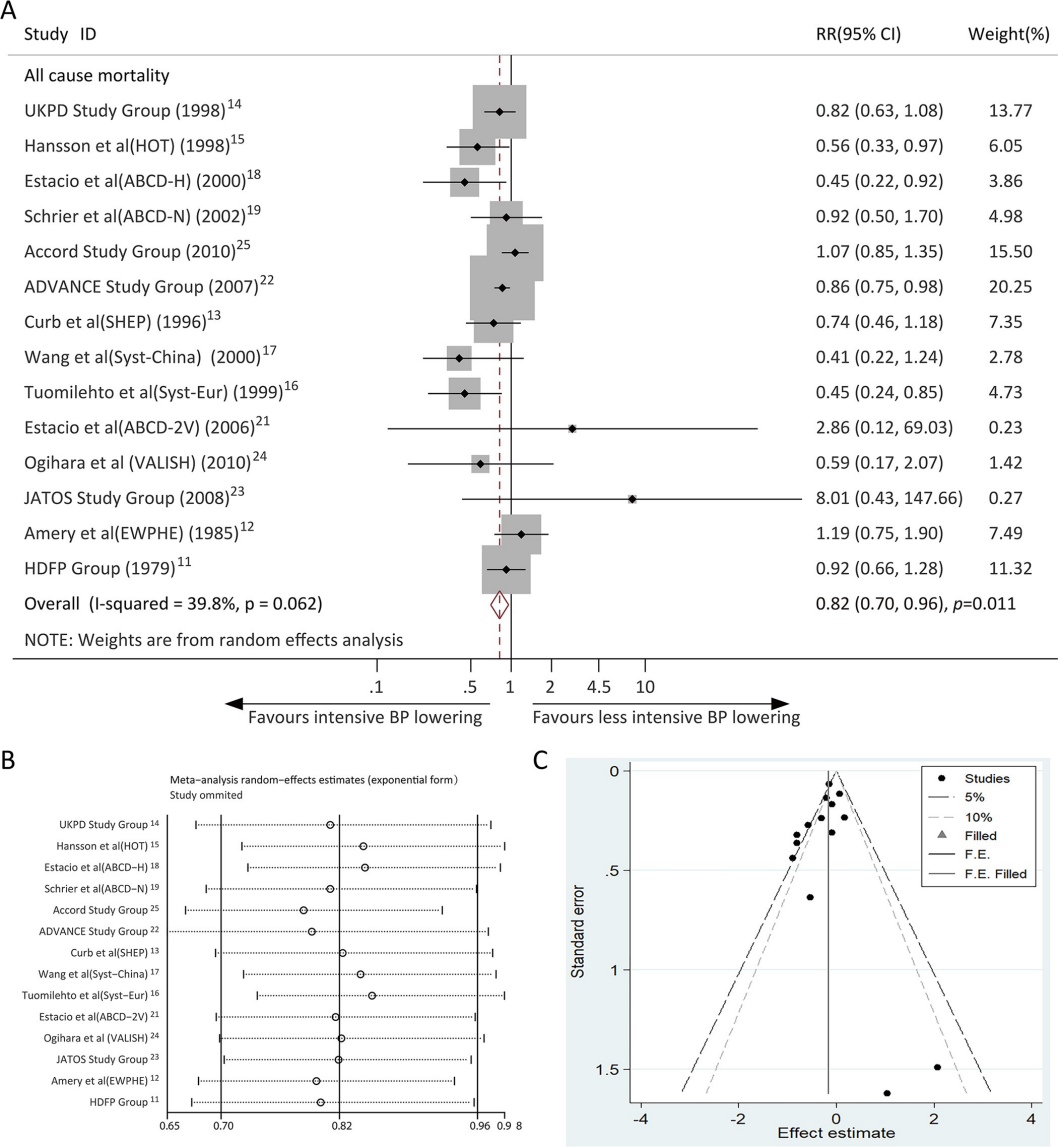
(A) Forest plot showing the effects of intensive versus less intensive blood pressure lowering treatment on all-cause mortality; (B) Plot of sensitivity analysis by excluding one study each time and the pooling estimate for the rest of the studies; (C) Funnel plot of publication bias test.

Data on the effect of intensive BP control on all-cause mortality were available from 14 trials. Overall, the RR for all-cause mortality among patients in the intensive BP lowering group was 0.82 (95% CI, 0.70–0.96; *P* = 0.011) compared with the less intensive BP lowering group; due to evidence of moderate heterogeneity (*I*^2^ = 39.8%, *P* for heterogeneity = 0.062; Fig 2A), the random-effects model was used. A sensitivity analysis was performed by excluding one trial each time and recalculating the pooled RR for the remaining trials, and none of the individual trials had an evident influence on the pooled effect size (Fig 2B). This analysis verified the robustness of the result. A visual inspection of the funnel plot revealed no evidence of publication bias (Fig 2C). Begg’s (*P* = 0.511) and Egger’s regression tests (*P* = 0.444) also indicated no publication bias in this meta-analysis.

### Effects of intensive BP lowering on CV outcomes

**Fig 3:**
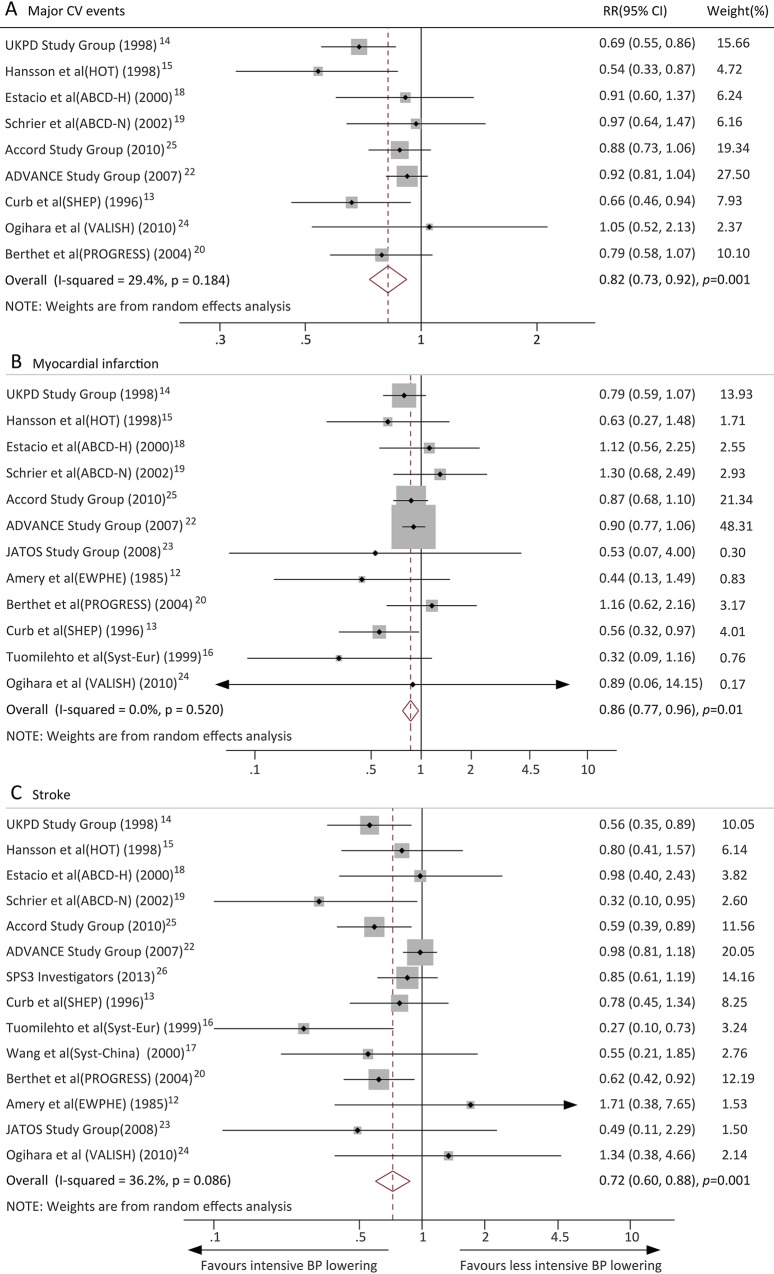
Effects of intensive blood pressure lowering on risk of cardiovascular outcomes (A) Major cardiovascular events; (B) Myocardial infarction; (C) Stroke.

**Fig 4:**
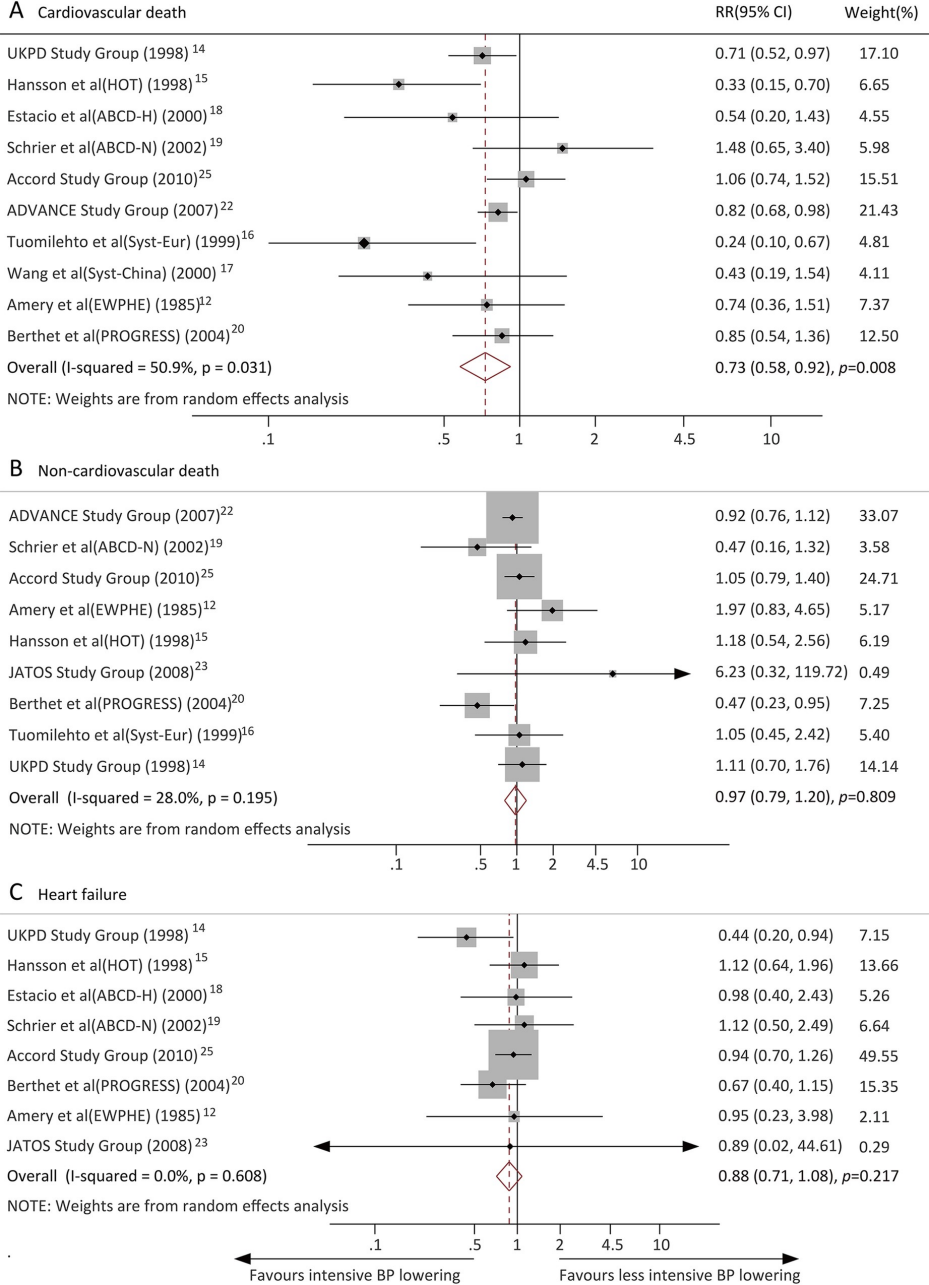
Effects of intensive blood pressure lowering on risk of cardiovascular outcomes (A) Cardiovascular death; (B) Non-cardiovascular death; (C) Heart failure.

Data regarding the effects of intensive BP lowering on major CV events were available from 9 trials. Overall, intensive BP lowering was associated with a significantly lower risk of major CV events (RR, 0.82, 95% CI, 0.73–0.92, *P* = 0.001; *I*^2^ = 29.4%) (Fig 3A). Twelve trials reported the MI outcome, and intensive BP lowering was associated with a 14% (RR, 0.86, 95% CI, 0.77–0.96, *P* = 0.01; *I*^2^ = 0.0%) (Fig 3B) reduction in the MI risk compared with less intensive BP lowering. Stroke was reported in 14 trials, and intensive BP lowering reduced the risk of stroke by 24% (RR, 0.72, 95% CI, 0.60–0.88, *P* = 0.001; *I*^2^ = 36.2%) (Fig 3C). Ten trials reported the CV death outcome. Compared with less intensive BP lowering, intensive BP lowering significantly reduced the risk of CV death (RR, 0.73, 95% CI, 0.58–0.92, *P* = 0.008; *I*^2^ = 50.9%) (Fig 4A). Nine trials reported the non-CV death outcome, and the risk of non-CV death was not reduced by intensive BP lowering (RR, 0.97, 95% CI, 0.79–1.20, *P* = 0.809; *I*^2^ = 28.0%) (Fig 4B). HF was reported in 8 trials, with no reduction in this outcome in patients allocated to the intensive BP lowering group compared with the less intensive BP lowering group (RR, 0.88, 95% CI, 0.71–1.08, *P* = 0.217; *I*^2^ = 0.0%) (Fig 4C).

### Effects of intensive BP lowering on renal outcomes

**Fig 5:**
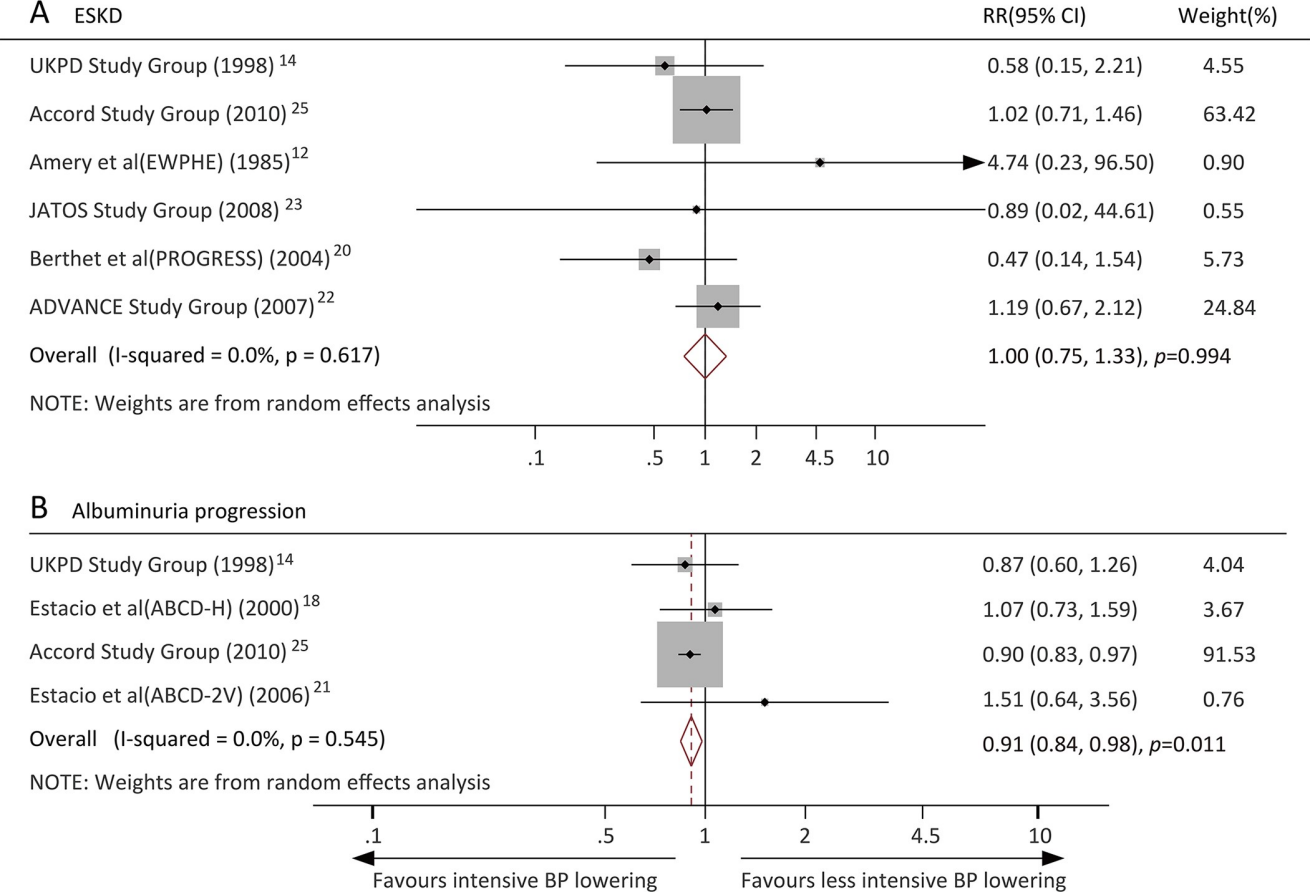
Effects of intensive blood pressure lowering on risk of renal outcomes (A) End stage kidney disease; (B) Albuminuria progression.

Six trials reported the ESKD outcome, and the pooled result showed no clear benefit for intensive BP lowering on the risk of ESKD compared with less intensive BP lowering (RR, 1.00, 95% CI, 0.75–1.33, *P* = 0.994; *I*^2^ = 0.0%) (Fig 5A). Five trials reported data about albuminuria progression and showed that intensive BP lowering reduced the risk of albuminuria progression by 9% (RR, 0.91, 95% CI, 0.84–0.98, *P* = 0.011; *I*^2^ = 0.0%) (Fig 5B).

### Subgroup analysis and meta-regression

**Fig 6:**
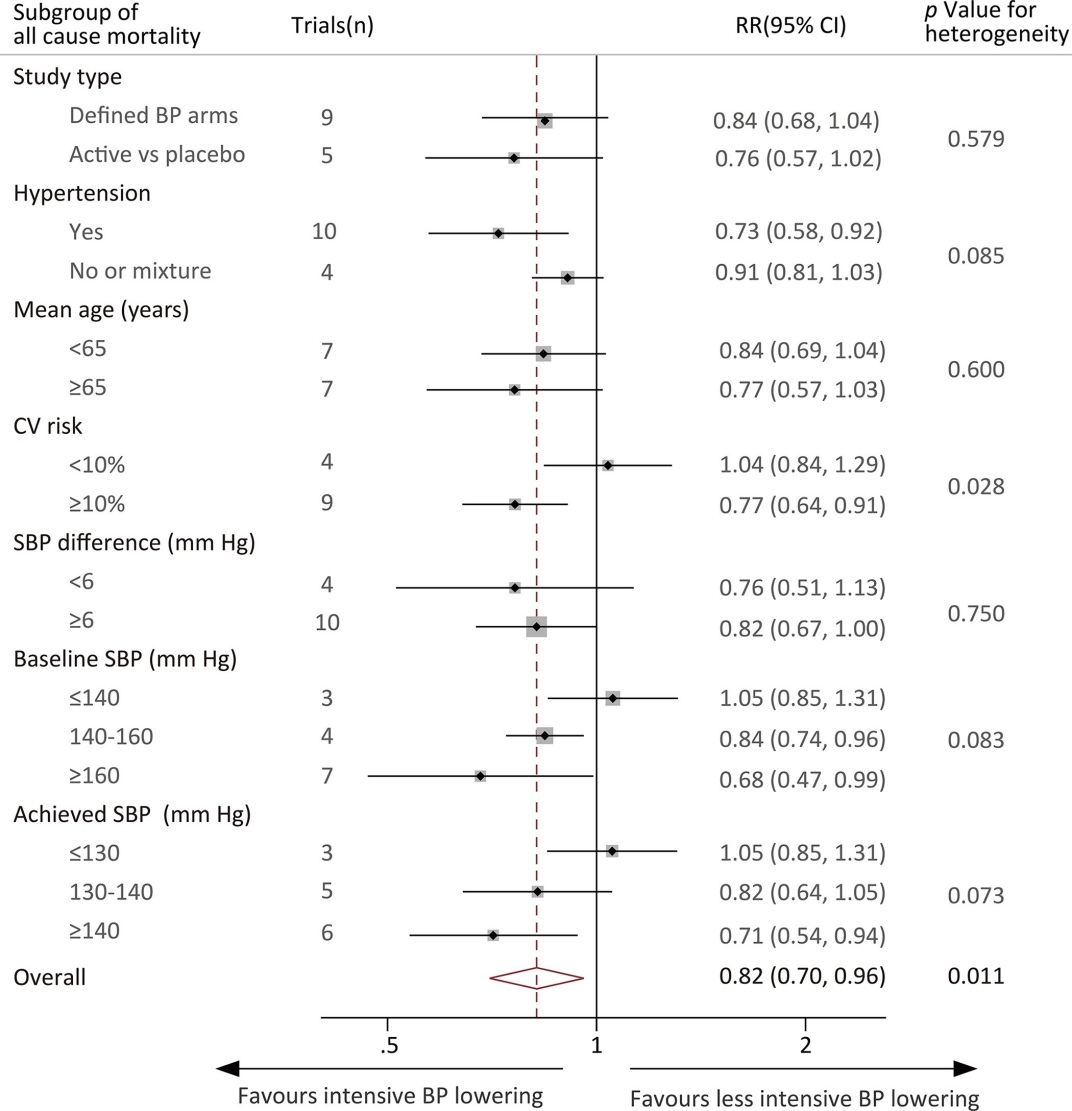
Effect of intensive blood pressure lowering on the risk of all-cause mortality in subgroups of trials.

The observed effects of intensive BP lowering treatment did not differ among most trial subgroups defined according to a broad range of baseline characteristics (*P* for heterogeneity *P*>0.05), including the type of treatment in the comparison arm (less intensive BP target or placebo), whether hypertension was present, mean age (<65 vs ≥65 years), baseline SBP of the entire cohort (<140 mm Hg vs 140–160 mm Hg vs >160 mm Hg), achieved SBP in the intensive BP lowering group (SBP<130 mm Hg vs SBP 130–140 mm Hg vs SBP ≥140 mm Hg) and SBP difference (<6 mm Hg vs ≥6 mm Hg) (Fig 6). In particular, there was no clear evidence that the benefits of intensive BP lowering varied by the mean baseline SBP of the trial participants or the mean achieved SBP in the intensive BP lowering group. However, in the subgroup stratified by CV risk, the result showed that in the trials that included patients with CV risks ≥10%, the RR of death in the intensive vs less intensive BP lowering arms was 0.77. The trials that included patients with CV risks <10% had an RR of 1.05. Formal testing for heterogeneity resulted in a *P* value of 0.028. Univariate meta-regression of intensive BP lowering on all-cause mortality according to the baseline characteristics also showed no evidence of heterogeneity (S2 Table).

## Discussion

This meta-analysis, which included 24,444 diabetic patients, demonstrates clear overall benefits for intensive BP lowering treatment. The risk of all-cause mortality was reduced by 18%, and the risks of most CV outcomes, including major CV events, MI, stroke and CV death, and albuminuria progression were also significantly reduced. However, there was no evidence to suggest that intensive BP lowering treatment reduced or increased the risk of non-CV death, HF or ESKD. The beneficial effect for all-cause mortality was consistent across most patient subgroups. Additionally, a significant benefit was achieved for those with baseline SBPs lower than 140 mm Hg and from further lowering the SBP to lower than 130 mm Hg. We also observed a possibility of a mortality benefit in trials that included patients with CV risks higher than 10% (*P* = 0.028).

Our study results are consistent with those of previous meta-analyses. In a meta-analysis of 8322 diabetic patients, Reboldi et al reported that tighter BP control could reduce the risk of stroke, but not the risk of MI, by 39%[28]. However, the meta-analysis by Reboldi et al included only five trials that aimed to evaluate the effects of more-tight BP control compared to less-tight BP control. In another meta-analysis aimed to assess the efficacy and safety of intensive BP lowering strategies among the general population, Xie et al. reported that compared to standard regimens, intensive BP lowering strategies reduced the risk of major CV events by 17% in diabetic patients[27]. The two meta-analyses assessed relatively fewer outcomes (major CV events, stroke and MI) that may show benefits from intensive BP lowering treatment. Our meta-analysis evaluated the effects of intensive BP lowering treatment on not only CV outcomes but also all-cause mortality and renal outcomes. We referred to a previous meta-analysis and used all-cause mortality as our primary outcome because this parameter balanced the competing risk of multiple clinical outcomes and because all-cause mortality was a “hard” outcome that was assessed similarly across studies[38]. As a whole, the results of our study and prior meta-analyses add to the body of evidence that diabetic patients may benefit more from intensive BP lowering treatment than less intensive BP lowering treatment.

The subgroup analysis of our study should be specifically noted. We observed that the diabetic patients with lower CV risk (<10%) showed no additional benefit, whereas the patients with higher CV risk (≥10%) showed an additional benefit (a 23% reduction in the risk of all-cause mortality) from intensive BP lowering treatment compared to less intensive BP lowering treatment. This finding was supported by a recent meta-analysis performed by Zanchetti *et al*.[39]. In this meta-analysis of 68 RCTs, the author reported that a 10/5 mm Hg SBP/diastolic BP (DBP) reduction reduced the incidence of major CV events by 0 (95% CI, -4 to 4), 9 (95% CI, 0 to 17), and 14 (95% CI, 5 to 26) events per 1000 patients treated for 5 years in the low-moderate (<5%), high (5%-10%) and very high CV risk (10%-20%) groups, respectively[39]. Recently, the SBP Intervention Trial (SPRINT) reported that intensive BP control (targeting an SBP of less than 120 mm Hg) compared with less intensive BP control (targeting an SBP of less than 140 mm Hg) could significantly reduce the risk of fatal and nonfatal major CV events and death from any cause among patients at high risk for CV events but without diabetes[40]. Our preliminary finding indicates that diabetic patients with higher CV risks may benefit more from intensive BP lowering treatment. These data will need to be reevaluated by further RCTs designed to assess the different effects of intensive BP lowering treatment on diabetic patients with different CV risks.

Currently, the optimal BP target for diabetic patients is under discussion. The latest Canadian and ACC/AHA guidelines both recommend that adult diabetic patients be treated to achieve <130/80 mm Hg[9, 10]. The subgroup analysis of our study showed that the beneficial effect for all-cause mortality was consistent in the patient subgroup stratified by the achieved SBP in the intensive BP lowering arms (<130 mm Hg vs 130–140 mm Hg vs ≥140 mm Hg) (*P* = 0.073 for heterogeneity). A previous meta-analysis performed by Brunström *et al*. showed that the anti-hypertensive treatment reduced the risk of stroke (a 35% reduction) but was not associated with a significant increase in all-cause and CV mortality, MI or HF if the SBP was lower or less than 130 mm Hg[30]. Our results and Brunström‘s results may provide some evidence for the adherence to the current recommendation of an achieved goal of <130/80 mm Hg for diabetic patients from the Canadian and ACC/AHA guidelines. However, the patients from most of the RCTs included in our or Brunström‘s subgroup analysis that were stratified to the achieved BP level (SBP< or >130 mm Hg) had a mean baseline BP level lower than 140 mm Hg or slightly higher than 140 mm Hg before treatment. These data may prevent the results of the subgroup analysis from being suitable for generalization to diabetic patients with high baseline BP levels (e.g., higher than 160 mm Hg). Thus, additional RCTs are needed to further assess the beneficial and harmful effects among diabetic patients with higher baseline BP levels who reach an achieved goal of <130/80 mm Hg.

This study had several strengths. First, we not only aimed to include more RCTs but also restricted our inclusion criteria to only include RCTs designed to evaluate the effects of intensive or active BP lowering treatment. Thus, our meta-analysis could provide more accurate evidence on the effects of intensive BP lowering treatment for diabetic patients. Second, we assessed mortality, which had obvious clinical importance and was similarly ascertained across studies and thus largely free of bias, as a hard clinical outcome. Third, we evaluated the effects of intensive BP lowering treatment on CV and renal outcomes. Therefore, we have provided a more comprehensive understanding of the overall effects of intensive BP lowering treatment among diabetic patients. Fourth, we used the CV risk to stratify the included RCTs. We found that diabetic patients with higher CV risk could benefit more from intensive BP lowering treatment than from less intensive BP lowering treatment. This finding is especially noteworthy. Fifth, we used rigorous methods, including sensitivity analysis, subgroup analysis and meta-regression, to assess the robustness of the study results.

Our study also had some limitations. First, despite considerable effort to contact the investigators of some of the trials, some investigators did not respond to us. Therefore, some trials were excluded due to a lack of data for the diabetic patients. Second, between-study variability, due to different patient characteristics and trial designs between the included studies, remained. The types of BP target (SBP or DBP) and specific target values varied across the included trials. Third, our study included fewer trials (16 trials) than the previous meta-analysis because of our strict inclusion criteria, and thus, the number of trials used to perform certain subgroup analyses was small (3 trials for the baseline BP level <140 mm Hg and 3 trials for the achieved BP level <130 mm Hg). This limitation should be considered when referring to our subgroup results. The number of included studies also limited the power for further exploration with multivariable meta-regression or multilevel subgroup analyses. Fourth, the lack of individual patient data, which would have allowed a sophisticated and more reliable assessment that accounted for patient characteristics or an analysis of BP levels within trials, was another limitation of our meta-analysis. Fifth, few studies included in our meta-analysis selectively reported adverse events, and the available data were too disparate to allow a formal meta-analysis.

## Conclusions

In conclusion, this systematic review and meta-analysis provides clear evidence of the benefits of intensive BP lowering treatment for type 2 diabetic patients. The results also provide some supporting evidence for the latest BP guideline of lowering BP to a goal of <130/80 mm Hg. More well-designed RCTs are needed to further evaluate the benefits or harms of a goal of <130/80 mm Hg with intensive BP lowering treatment.

## Supporting information

S1 Appendix Search strategies. Same search strategies were used in literature search in PubMed and Cochrane Library, EMBASE and Science Citation Index. Here, we provided the search strategies in PubMed and EMBASE.
(DOCX)

S1 Fig Summary for risk of bias of included trials. The green symbols represent low risk of bias, the yellow symbols represent unclear risk of bias, and the red symbols represent high risk of bias. The figure was generated using Review Manager Version 5.2.
(JPG)

S2 Fig Risk of bias graph of included trials. Each methodological quality item is presented as percentages across all included studies. The figure was generated using Review Manager Version 5.2.
(JPG)

S1 Table PRISMA checklist 2009. (DOC)

S2 Table Univariate meta-regression of intensive blood pressure lowering on all-cause mortality. (DOCX)
