# Supplementary material for: Effects of intensive blood pressure lowering on mortality and cardiovascular and renal outcomes in type 2 diabetic patients: A meta-analysis
Source: PLoS One. 2019 Apr 12;14(4):e0215362. doi: 10.1371/journal.pone.0215362 (PMC6461269; doi:10.1371/journal.pone.0215362)
Supplement: S1 Appendix — Same search strategies were used in literature search in PubMed and Cochrane Library, EMBASE and Science Citation Index. Here, we provided the search strategies in PubMed and EMBASE. (DOCX) [file pone.0215362.s001.docx]

**S1 Appendix. Search strategies**

**Same search strategies were used in literature search in PubMed and Cochrane Library, EMBASE and Science Citation Index. Here, we provided the search strategies in PubMed and EMBASE.**

**PubMed**

1. antihypertensive agents

2. chlorothiazide

3. chlorthalidone

4. hydralazine

5. hydrochlorothiazide

6. indapamide

7. minoxidil

8. losartan

9. imidazole

10. irbesartan

11. candesartan

12. eprosartan

13. valsartan

14. olmesartan

15. telmisartan

16. angiotensin converting enzyme inhibitors

17. captopril

18. enalapril

19. fosinopril

20. lisinopril

21. perindopril

22. ramipril

23. saralasin

24. teprotide

25. Angiotensin 2 Receptor Antagonist

26. Angiotensin Receptor Antagonist

27. Angiotensin II Antagonist

28. AT 2 receptor blocker

29. AT 2 receptor antagonist

30. angiotensin receptor antagonist

31. Calcium Channel Blockers

32. amlodipine

33. diltiazem

34. felodipine

35. nicardipine

36. nifedipine

37. nimodipine

38. nisoldipine

39. nitrendipine

40. verapamil

41. adrenergic beta-antagonists

42. alprenolol

43. atenolol

44. carvedilol

45. bisoprolol

46. metoprolol

47. nadolol

48. oxprenolol

49. pindolol

50. propranolol

51. adrenergic alpha-antagonists

52. labetalol

53. prazosin

54. diuretics

55. spironolactone

56. triamterene

57. bumetanide

58. furosemide

59. or/1-58

60. clinical and trial

61. randomized and controlled and trial

62. random and allocation

63. single blind and method

64. double blind and method

65. or/60-64

66. target level

67. target blood pressure

68. Target systolic blood pressure

69. Target diastolic blood pressure

70. Intensive treatment

71. Intensive blood pressure treatment

72. Intensive antihypertensive treatment

73. Intensive control

74. Intensive blood pressure control

75. Tight control

76. Tight blood pressure control

77. Strict control

78. Strict blood pressure control

79. active treatment

80. active blood pressure treatment

81. active antihypertensive treatment

82. active control

83. active blood pressure control

84. or/66-83

85. child

86. retrospective study

87. editorial

88. review

89. meta analysis

90. or/85-89

91. #59 and #65 and #84

92. #91 not #90

**EMBASE**

1. antihypertensive agents

2. chlorothiazide

3. chlorthalidone

4. hydralazine

5. hydrochlorothiazide

6. indapamide

7. minoxidil

8. losartan

9. imidazole

10. irbesartan

11. candesartan

12. eprosartan

13. valsartan

14. olmesartan

15. telmisartan

16. angiotensin converting enzyme inhibitors

17. captopril

18. enalapril

19. fosinopril

20. lisinopril

21. perindopril

22. ramipril

23. saralasin

24. teprotide

25. Angiotensin 2 Receptor Antagonist

26. Angiotensin Receptor Antagonist

27. Angiotensin II Antagonist

28. AT 2 receptor blocker

29. AT 2 receptor antagonist

30. angiotensin receptor antagonist

31. Calcium Channel Blockers

32. amlodipine

33. diltiazem

34. felodipine

35. nicardipine

36. nifedipine

37. nimodipine

38. nisoldipine

39. nitrendipine

40. verapamil

41. adrenergic beta-antagonists

42. alprenolol

43. atenolol

44. carvedilol

45. bisoprolol

46. metoprolol

47. nadolol

48. oxprenolol

49. pindolol

50. propranolol

51. adrenergic alpha-antagonists

52. labetalol

53. prazosin

54. diuretics

55. spironolactone

56. triamterene

57. bumetanide

58. furosemide

59. or/1-58

60. clinical and trial

61. randomized and controlled and trial

62. random and allocation

63. single blind and method

64. double blind and method

65. or/60-64

66. target level

67. target blood pressure

68. Target systolic blood pressure

69. Target diastolic blood pressure

70. Intensive treatment

71. Intensive blood pressure treatment

72. Intensive antihypertensive treatment

73. Intensive control

74. Intensive blood pressure control

75. Tight control

76. Tight blood pressure control

77. Strict control

78. Strict blood pressure control

79. active treatment

80. active blood pressure treatment

81. active antihypertensive treatment

82. active control

83. active blood pressure control

84. or/66-83

85. #59 and # 65 and # 84
