# Supplementary figures and images for: Effects of intensive blood pressure lowering on mortality and cardiovascular and renal outcomes in type 2 diabetic patients: A meta-analysis
Source: PLoS One. 2019 Apr 12;14(4):e0215362. doi: 10.1371/journal.pone.0215362 (PMC6461269; doi:10.1371/journal.pone.0215362)

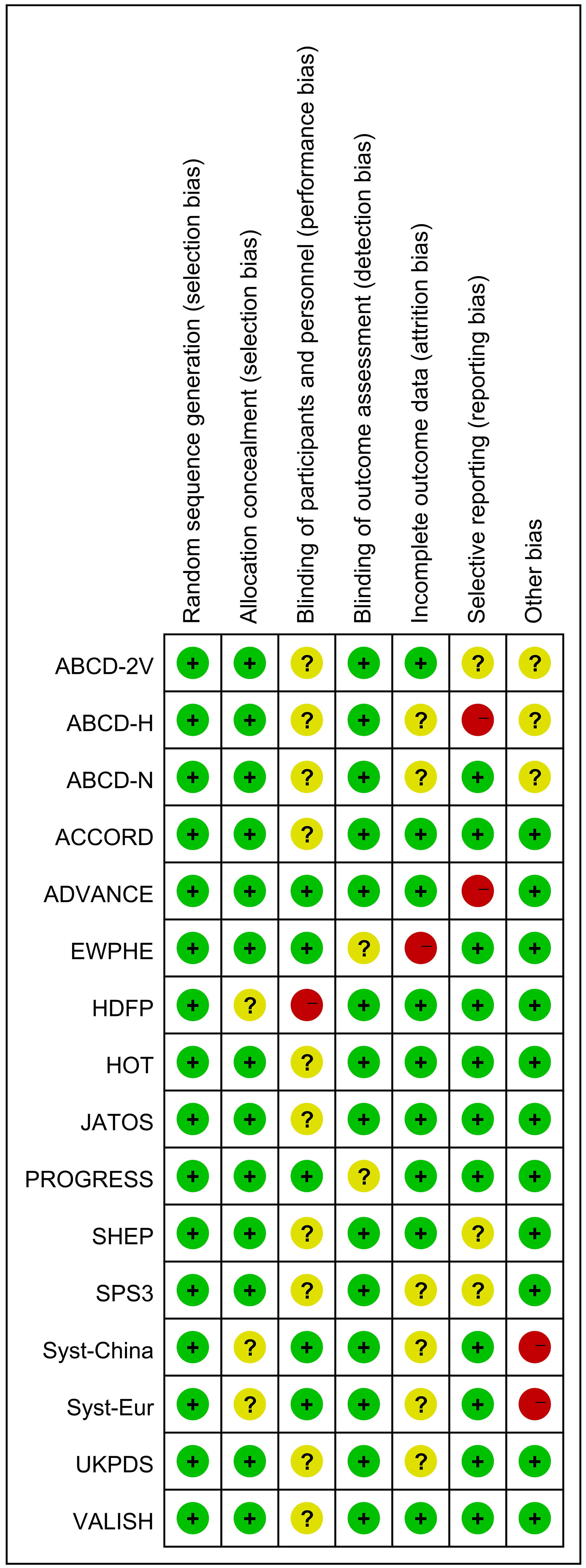

Supplement: S1 Fig — The green symbols represent low risk of bias, the yellow symbols represent unclear risk of bias, and the red symbols represent high risk of bias. The figure was generated using Review Manager Version 5.2. (JPG) [file pone.0215362.s002.jpg]

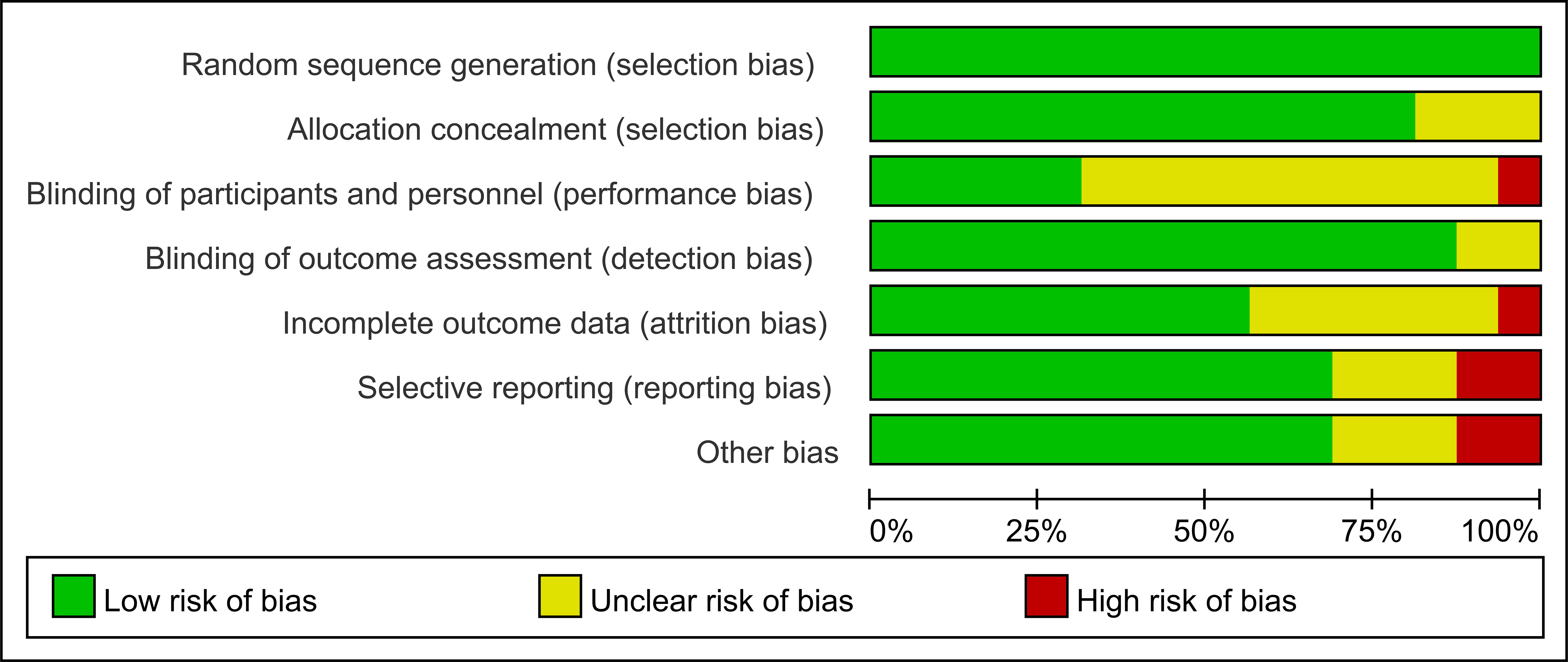

Supplement: S2 Fig — Each methodological quality item is presented as percentages across all included studies. The figure was generated using Review Manager Version 5.2. (JPG) [file pone.0215362.s003.jpg]
