# Supplementary material for: Effects of intensive blood pressure lowering on mortality and cardiovascular and renal outcomes in type 2 diabetic patients: A meta-analysis
Source: PLoS One. 2019 Apr 12;14(4):e0215362. doi: 10.1371/journal.pone.0215362 (PMC6461269; doi:10.1371/journal.pone.0215362)
Supplement: S2 Table — (DOCX) [file pone.0215362.s005.docx]

**S2 Table Univariate Meta-regression of Intensive Blood Pressure Lowering on All-cause Mortality**

| **Variable** | **Studies** | **Scale** | **Proportion change in**  **RR and 95%CI** | ***p*-value** |
| --- | --- | --- | --- | --- |
| Age | 14 | per year | 0.9969(0.9666-1.0282) | 0.831 |
| Patient number | 14 | per 100 | 1.0017(0.9965-1.0068) | 0.495 |
| Median follow-up years | 14 | per year | 1.0508(0.9253-1.1932) | 0.413 |
| Baseline systolic BP | 14 | per 1 mmHg | 0.9937(0.9825-1.0050) | 0.249 |
| Cardiovascular risk | 13 | Per 1% | 0.9853(0.9671-1.0038) | 0.107 |

RR: relative risk; CI: confidence interval
